# Health Conditions in Tehran’s Girl Schools from 1927 to 1934: A Brief Survey

**DOI:** 10.34172/aim.2023.19

**Published:** 2023-02-01

**Authors:** Behzad Karimi

**Affiliations:** ^1^Department of Iranian Studies, Meybod University, Yazd, Iran

**Keywords:** Health, Iran, Modernization, Schools, Tehran

## Abstract

A new model of relations emerged in schools after the establishment of new educational centers in Iran in the mid-Qajar era. The cultural authorities of the time were particularly interested in school health, which was adopted as a principle from the European, particularly French, school systems. During the period 1925 to 1941, with special attention to the new Western models of education, school health changed more and more. This study provides a descriptive-analytical report on state health policies in girl schools in Tehran, Iran, from 1927 to 1934, based on health records available at the National Archives of Iran. The findings reveal that since the mid-first Pahlavi era, officials from the Ministry of Science became increasingly involved in the issue of health, which resulted in institutionalization of health and medical examination of students, establishment of the School Health Office, publication of theoretical health discussions in magazines, and teaching of health principles to students, thereby improving the level of health in Tehran schools during the study period. The study aims to provide Iranian physicians and health policymakers with a review of this historical experience.

## Introduction

 During the Qajar period (1789–1925), Iran suffered from severe epidemics such as the plague and cholera. Numerous records indicate that despite the considerable reforms by *Amir Kabir (1807–1852)* and *Mirza Hossein Khan Sepahsalar (1828–1881)*, the country’s health status at the time was dreadful and grave challenges affected individual and public health.^1^ Although there were no large-scale epidemics after Reza Shah’s rise to power (1925), various illnesses including syphilis, chlamydia trachomatis, tuberculosis, leprosy, *pediculus humanus capitis*, *pediculus humanus corporis*, *alopecia areata*, dysentery, atopic dermatitis, typhoid, meningitis, and diphtheria were widespread, occasionally infecting school children.^2^ Politicians of the period sought to modernize health and medicine in order to improve the Iranian situation and create a productive and healthy population, which is the basis of any modern society. This started with focusing on school health as the first step in the socialization of children and adolescents.^3^ The first Pahlavi government addressed the issue by enacting several specific initiatives. First, several health education textbooks for girls as future mothers were translated and written. A course on child health and nurturing was taught in girl schools throughout elementary school. Some of the covered topics included diseases, germs, ways to deal with their effects, disinfection, air conditioning, the dangers of dust, light and its importance for health, the conditions of drinking water and finally, body and home cleansing. It also covered topics like infant care, clothes and crib hygiene, baby food, breastfeeding, infant growth, vaccination, tooth health, walking, and weaning related to child-rearing.^4^ Second, the school health department was founded within the Ministry of Science. This department was responsible for inspecting schools on a regular basis with the assistance of qualified personnel or experienced physicians and providing reports that would serve as a reference for health policymakers to develop school health improvement initiatives.^5^

 School health has significantly improved in Iran over the last several decades. Still, many challenges linger even today. The COVID-19 pandemic once again pointed to school health as a key area of investigation.^6^ Also, we have reports showing that various diseases are prevalent among students in different parts of Iran, which spread through schools. Some of these diseases are the same diseases that were prevalent among students during the first Pahlavi era.^7^ This study reviews an invaluable historical experience that can benefit contemporary physicians and health decision-makers in Iran.

## Theoretical Foundations of School Health

**Figure 1:**
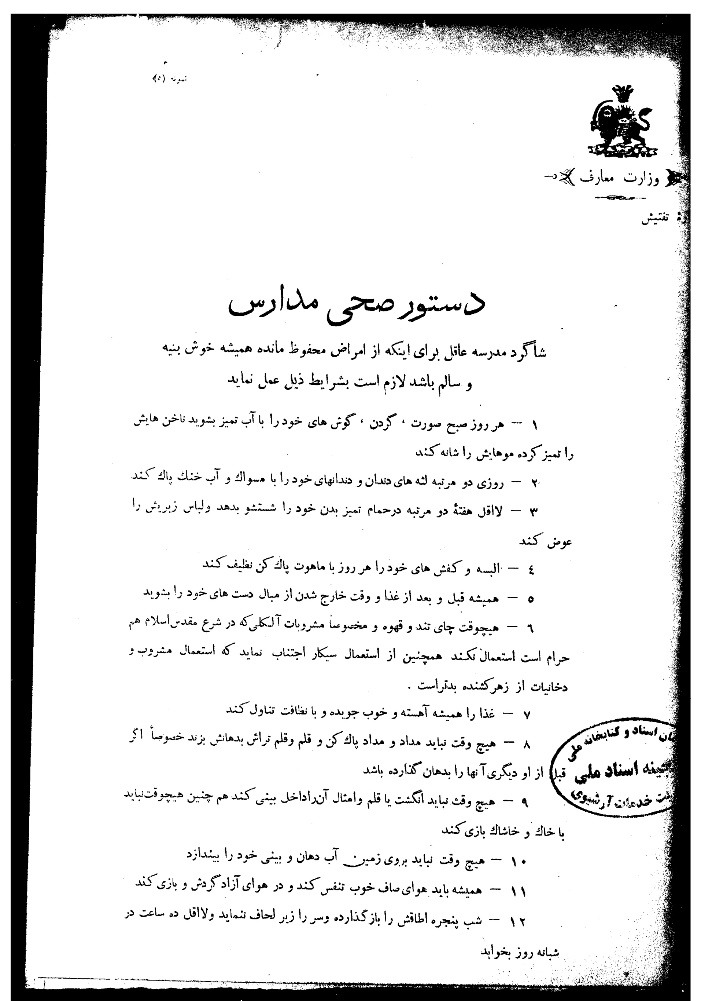
School Health Instructions, Issued by the Inspection Office Affiliated to Ministry of Sciences.

 The theoretical foundations of school health by adopting European and American models, particularly since the beginning of the first Pahlavi era (1925), drew the attention of Iranian physicians. A document (i.e. School Health Instructions), dated 1924, shows that cultural authorities heeded the theoretical discussions on student health even before the first Pahlavi era. This document contains simple health instructions for students specified in 12 separate paragraphs, including hand washing, bathing, and avoiding coffee or alcoholic beverages.^8^ Such statements reveal a clear sense of translation (Figure 1). It is not far-fetched to argue that the health policies of the Ministry of Science were based on the theories and experiences of European countries in both theory and practice. A cultural thinker, *Mahmoud Khan Shimi*, noted in an article, “For nearly 20 years, great attention has been given to children’s health in the European and American schools with attempts to improve it every year, eliminate hazards, and make tools of physical and spiritual development more easily accessible”.^9^ He then voices his concern about the lack of attention to students’ health by drawing a grim picture of Tehran’s schools, likening children’s condition to that of animals. He then discusses different issues concerning school health in different sections. The first section of his paper covers topics such as the suitability of school buildings, classroom capacity in accordance with the French law, ventilation, and heating system. The second section, “student health,” delves into sleep hours, rest intervals, exercise, and homework. Finally, in a section entitled “medical examination,” *Shimi* discusses infectious diseases and highlights the necessity of frequent medical examinations for students, suggesting a medical examination model that is comparable to that used in the United States and Europe.^9^ *Alikhan Isfahani* (*Hakim Azam*), also known as *Parto*, who was the Deputy Minister of Health in 1925, draws great attention to school health in a meticulously written article entitled “A few words on health in elementary schools.” According to this article, school health was a top concern in national health management. He introduces the condition of students as pitiful, “Most of our children are deprived of a healthy temperament and physical health. They are not sound in the proper sense. If you examine these schools from a medical perspective, you will see pale cheeks, throat congestion, hunched backs, sad faces, and thousands of other symptoms, and you will quickly conclude that the majority of these children have chronic diseases. You will be compelled to admit that most of our children do not have a healthy life”.^10^ *Alikhan* then addresses the various aspects of health in elementary students under three headings: food and drink, socializing, and hobbies, and asks their parents to engage in implementing health principles actively.^10^ The significance of theoretical discussions on school health in the early Pahlavi era is highlighted by the fact that subsequent measures taken by the Ministry of Science for the inspection of school health and health principles were based primarily on the same guidelines, as confirmed by the checklist of its inspectors from the following years. It seems that the theoretical discussions about student health grew more extensive and detailed as medical and cultural authorities became more aware of the benefits of school health and current practices in Europe and the United States.^11^

## Documents Analysis

 According to *Shimi*, health monitoring of schools in Tehran, as well as medical inspections of students, started during *Ahmad Badr*’s (*Nasir al- Dawla*) tenure as Minister of Science.^9^ Based on his narrative, and given the publishing date of his article in the Journal of Education (*Talim Va Tarbiat*) in 1925, health monitoring and medical examination of students in Tehran dates back to 1917. Unfortunately, there are no reports available about the results of these inspectors’ or physicians’ actions during this or the following years until 1927. The earliest weekly or monthly inspection reports by the Ministry of Science date back to 1927.

 The National Archives of Iran holds several documents on the school modernization project, most of which pertain to girl schools in Tehran from 1927 to 1934. Given the patriarchal foundation of Iranian culture at the time, female students were in a critical condition. Even the permissibility of their education in modern schools was subject to question in this traditional society. Girls constantly faced severe health challenges due to pregnancy, sexually transmitted diseases (which sometimes were not even referred to experts), and their responsibility of taking care of children. In addition, the dress code (at least until the unveiling policy in 1935) exposed girls more than male students to greater risks of skin and hair diseases, which used to be quite common. Also, there were fewer female students and girl schools in Tehran compared to boy schools. Therefore, the assessment of the health conditions in these schools was more accessible and concise.

 The available documents indicate that there were at least 58 girl schools with 2057 students in Tehran during the research period. A total of 48 of these were elementary schools, and the rest were high schools.^12^ The documents reviewed here are all related to girls’ elementary schools in Tehran.

 It should be mentioned that the accuracy of the contents of these reports varies depending on the source of the report. Obviously, greater caution must be taken when relying on school authorities’ reports. Meanwhile, the School Health Office reports appear to be more reliable due to the inspectors’ expertise, scientific sampling methods, and the more specialized content in the reports.

**Figure 2:**
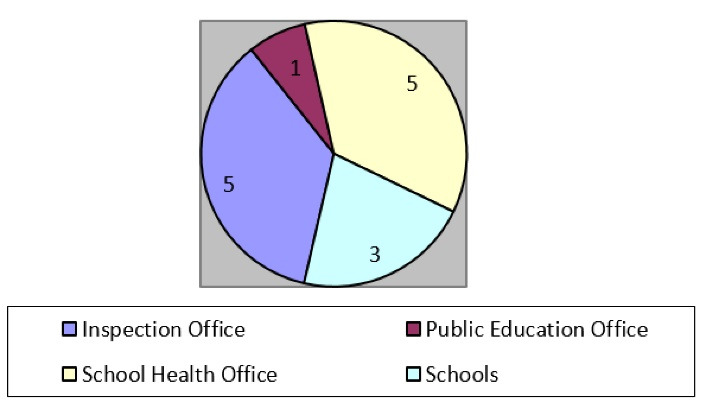
Frequency of Health Reports from Tehran's Girl Schools 1927–1934, Based on the Issuing Authorities and the Documents Reviewed in this Article.

 Secondly, health inspection makes up only part of the content of reports prepared by the Inspection and Public Education offices. According to the documents, the Ministry of Science established a “School Health Office” in 1931, which directly prepared specialized health reports about students and schools; this is another indicator of the health priority for Ministry of Science officials (Figure 2).

## Health Condition in Tehran’s Girl Schools from 1927 to 1934

 The medical examination of girl schools was performed weekly and monthly, and the results of the inspections by the Inspection Office’s inspectors or physicians were reported to the Inspection Office’s or School Health Office’s director. A review of nine weekly, monthly, or school reports reveals important insights. It should be noted that the inspectors’ reports seem to be inadequate or biased in some cases.

**Figure 3:**
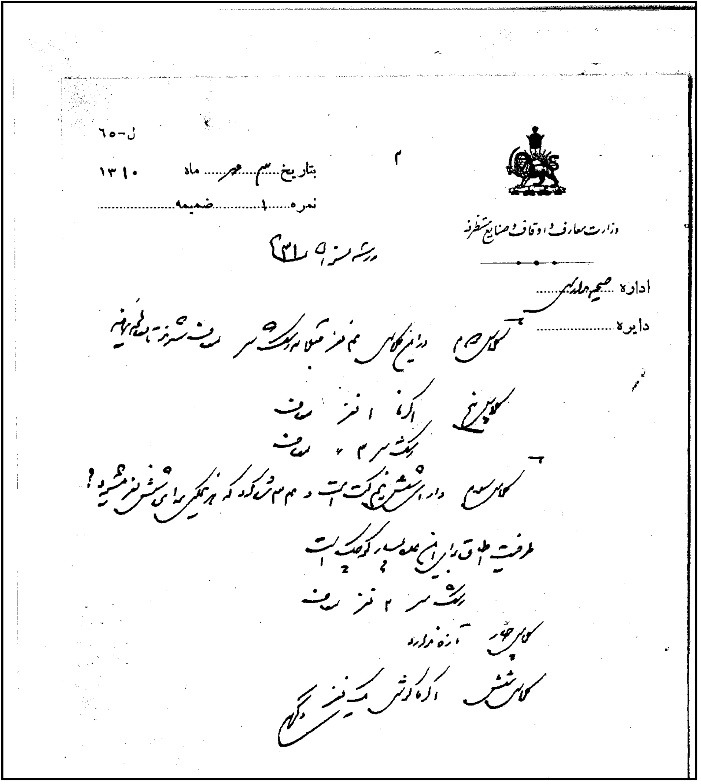
A health Report Related to the Tehran's Girl Schools, Issued by the School Health Office.

**Figure 4:**
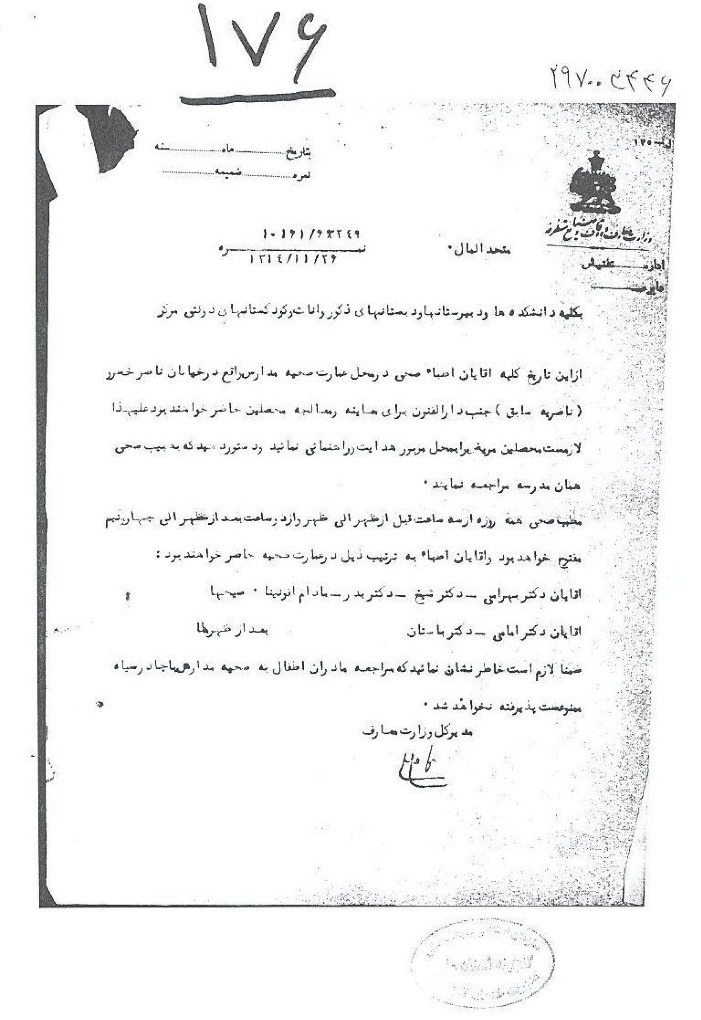
A Circular that Required the Examination of Students at the School Health Office.

 Inspectors visited schools on a regular basis and prepared reports. A review of the health sections of the reports indicates that, although the inspectors were not physicians, they were adequately trained to identify common diseases, as evidenced by the fact that they identified and reported such cases by specifying the names and types of diseases. Furthermore, physicians commissioned by the Inspection Office or the Health Office recorded students’ health conditions as well as the environmental health of schools (Figure 3).^13^ After registering the number of absent ill students, the inspectors and physicians would undertake clinical examinations on them at their homes. If infectious diseases were diagnosed, the students were either separated from others or required not to attend school until they were cured.^14^ Interestingly, the state of the school building and the proportion of classroom capacity to student population were meticulously noted in these reports, and any violations were reported to the director of the Inspection Office or the School Health Office.^15^ Another noteworthy point is that, in the final years of the first Pahlavi era, authorities from the Ministry of Science used the capabilities of medical schools to specialize in health examinations. According to a Health Office document dated April 1935, several final-year ophthalmology students from the Tehran medical school, led by Professor *Mohammad Gholi Shams (1905–1996)*, were introduced to the head of the Inspection Office to perform administrative tasks and specialized eye examinations. *Shams* was the founder of modern ophthalmology in Iran and was later dubbed the father of Iranian ophthalmology.^16^ Per the letter, authorities from the Health Office insisted on performing tests at the start of the (solar) year and had designated different schools for each of the six students introduced. Such measures may have contributed to the establishment of modern medical schools in Iran.^17^ According to the document shown in Figure 4, we now know that in addition to *Professor Shams*, other persons cooperated with the Health Office regarding the routine examination of students. These included *professor Hossein Khan Bahrami (1876–1941),* one of the pioneers of Iran’s modern health, *Professor Mohammad Sheikh (1868-1938),* a well-known anatomy professor at *Dar al- Fonun*, *Professor Ahmad Emami*, skin and venereal diseases specialist, *Madame Afunina*, an unknown ophthalmologist, and professor *Nosrat al-Allah Bastan (1903-1977),* a famous ophthalmologist.^3^

 The type of diseases and their frequency are the next notable aspects that may be uncovered through the study of health reports. Chronic skin diseases, chronic infections, and bacterial infections, all of which resulted from inadequate personal and public hygiene, were among the most common infectious diseases in Tehran’s girl schools, according to a review of reports dating from 1927 to 1934.

 The information extracted from the documents shows that the most common diseases were cutaneous leishmaniasis, chlamydia trachomatis, atopic dermatitis, nits, impetigo, *pediculus humanus capitis*, facial boils, and otitis. Therefore, school health authorities would provide recommendations to students on avoiding or treating such diseases.

 Examining and treating students were not the only tactics used in schools to prevent diseases. Preventative measures were also on the agenda. One of these solutions was immunizing students with vaccinations. Vaccination started in Iran at the beginning of the Qajar period with the smallpox vaccine and has continued since then.^18^ By the end of the Qajar period, the Pasteur Institute was established at the order of *Ahmad Shah Qajar (1909–1925)* to produce vaccines. This institute enabled the vaccination program in schools, including girl schools, during the first Pahlavi era.^19^

 At the end of the period studied in this article, important measures were taken to institutionalize hygiene in schools. The establishment of the Health Office in 1931 and the issuing of bylaws concerning the medical examination of children and students in the school health center on Nasser Khosrow Street by the Inspection Office of the Ministry of Science in February 1937 to all public colleges, high schools, elementary schools, and kindergartens in the capital prove the institutionalization of school health affairs in the first Pahlavi era.^20^ The review results also show that the Khiaban-e Mashin girl school with 30 sick students and Esmatieh and No. 15, with two sick students, had the highest and lowest records.^21^

## Conclusion

 Using documents from the National Archives of Iran and other reliable sources, this article attempted to review the experience of health modernization in girl schools in Tehran from 1927 to 1934. Content analysis of documents recording the type and frequency of diseases, student health conditions, and environmental health conditions of schools reveals the disorder of health affairs, the prevalence of various diseases among female students, and the seriousness of the health situation in the early Pahlavi era. The findings also reveal that, since the mid-first Pahlavi era, officials from the Ministry of Science, in order to improve the Iranian race and create a productive and healthy population, became increasingly involved in the issue of health in schools, which resulted in the institutionalization of health and medical examination of students, the establishment of the School Health Office, the publication of theoretical health discussions in magazines, and the teaching of health principles to students, thereby improving the level of health in Tehran schools during the study period.
